# Global Prevalence of Aspirin Use for Primary Prevention of Cardiovascular Disease: A Cross-Sectional Study of Nationally Representative, Individual-Level Data

**DOI:** 10.5334/gh.1323

**Published:** 2024-05-03

**Authors:** Sang Gune K. Yoo, Grace S. Chung, Silver K. Bahendeka, Abla M. Sibai, Albertino Damasceno, Farshad Farzadfar, Peter Rohloff, Corine Houehanou, Bolormaa Norov, Khem B. Karki, Mohammadreza Azangou-Khyavy, Maja E. Marcus, Krishna K. Aryal, Luisa C. C. Brant, Michaela Theilmann, Renata Cífková, Nuno Lunet, Mongal S. Gurung, Joseph Kibachio Mwangi, Joao Martins, Rosa Haghshenas, Lela Sturua, Sebastian Vollmer, Till Bärnighausen, Rifat Atun, Jeremy B. Sussman, Kavita Singh, Sahar Saeedi Moghaddam, David Guwatudde, Pascal Geldsetzer, Jennifer Manne-Goehler, Mark D. Huffman, Justine I. Davies, David Flood

**Affiliations:** 1 Cardiovascular Division, Department of Internal Medicine, Washington University in St Louis, St Louis, MO, USA; 2 Department of Internal Medicine, University of Michigan, Ann Arbor, MI, USA; 3 Department of Internal Medicine, MKPGMS Uganda Martyrs University, Kampala; 4 St Francis Hospital, Nsambya, Kampala, Uganda; 5 Epidemiology and Population Health Department, Faculty of Health Sciences, American University of Beirut, Beirut, Lebanon; 6 Faculty of Medicine, Eduardo Mondlane University, Maputo, Mozambique; 7 Nucleo de Investigaçao, Departamento de Medicina, Hospital Central do Maputo, Maputo Mozambique; 8 Non-Communicable Diseases Research Center, Endocrinology and Metabolism Population Sciences Institute, Tehran University of Medical Sciences, Tehran, Iran; 9 Center for Indigenous Health Research, Wuqu’Kawoq, Tecpán, Guatemala; 10 Division of Global Health Equity, Brigham and Women’s Hospital, Boston, MA, USA; 11 Laboratory of Epidemiology of Chronic and Neurological Diseases, Faculty of Health Sciences, University of Abomey-Calavi, Cotonou, Benin; 12 Nutrition Department, National Center for Public Health, Ulaanbaatar, Mongolia; 13 Department of Community Medicine and Public Health, Institute of Medicine, Tribhuvan University, Kathmandu, Nepal; 14 Division of Infectious Diseases, Brigham and Women’s Hospital, Harvard Medical School, Boston, MA, USA; 15 Bergen Centre for Ethics and Priority Setting in Health, Department of Global Public Health and Primary Care, University of Bergen, Bergen, Norway; 16 Public Health Promotion and Development Organization, Kathmandu, Nepal; 17 Departamento de Clínica Médica, Universidade Federal de Minas Gerais, Belo Horizonte, Minas Gerais, Brazil; 18 Telehealth Center, Hospital das Clínicas da Universidade Federal de Minas Gerais, Belo Horizonte, Brazil; 19 Heidelberg Institute of Global Health, Faculty of Medicine and University Hospital, Heidelberg University, Heidelberg, Germany; 20 Professorship of Behavioral Sciences in Prevention and Care, Technical University of Munich, Germany; 21 Center for Cardiovascular Prevention, First Faculty of Medicine and Thomayer University Hospital, Charles University in Prague, Prague, Czechia; 22 Department of Medicine II, First Faculty of Medicine, Charles University in Prague, Prague, Czechia; 23 Department of Public Health and Forensic Health Sciences and Medical Education, Faculty of Medicine, University of Porto, Porto, Portugal; 24 EPIUnit –Institute of Public Health, University of Porto, Porto, Portugal; 25 ITR –Laboratory for Integrative and Translational Research in Population Health, Porto, Portugal; 26 Health Research and Epidemiology Unit, Ministry of Health, Thimphu, Bhutan; 27 Division of Non-Communicable Diseases, Ministry of Health, Nairobi, Kenya; 28 The Institute of Global Health, Faculty of Medicine, University of Geneva, Geneva, Switzerland; 29 Faculty of Medicine and Health Sciences, Universidade Nacional Timor Lorosa’e, Dili, Timor-Leste; 30 Non-Communicable Disease Department, National Center for Disease Control and Public Health, Tbilisi, Georgia; 31 Public Health Department, Petre Shotadze Tbilisi Medical Academy, Georgia; 32 Department of Economics & Centre for Modern Indian Studies, University of Goettingen, Göttingen, Germany; 33 Harvard Center for Population and Development Studies, Harvard University, Cambridge, USA; 34 Africa Health Research Institute, Somkhele and Durban, South Africa; 35 Department of Global Health and Population, Harvard T.H. Chan School of Public Health, Harvard University, Boston, MA, USA; 36 Department of Global Health and Social Medicine, Harvard Medical School, Harvard University, Boston, MA, USA; 37 Center for Clinical Management Research, VA Ann Arbor Healthcare System, Ann Arbor, MI, USA; 38 Centre For Chronic Disease Control, New Delhi, India; 39 Endocrinology and Metabolism Research Center, Endocrinology and Metabolism Clinical Sciences Institute, Tehran University of Medical Sciences, Tehran, Iran; 40 Kiel Institute for the World Economy, Kiel, Germany; 41 Department of Epidemiology and Biostatistics, School of Public Health, Makerere University, Kampala, Uganda; 42 Division of Primary Care and Population Health, Stanford University, USA; 43 Chan Zuckerberg Biohub –San Francisco, San Francisco, California, USA; 44 Medical Practice Evaluation Center, Massachusetts General Hospital, Harvard Medical School, Boston, MA, USA; 45 Department of Medicine and Global Health Center, Washington University in St Louis, St Louis, MO, USA; 46 Department of Preventive Medicine, Northwestern University Feinberg School of Medicine, Chicago, IL, USA; 47 The George Institute for Global Health, University of New South Wales, Sydney, Australia; 48 Institute for Applied Health Research, University of Birmingham, UK; 49 Centre for Global Surgery, Department of Global Health, Stellenbosch University, Cape Town, South Africa; 50 Medical Research Council/Wits University Rural Public Health and Health Transitions Research Unit, Faculty of Health Sciences, School of Public Health, University of the Witwatersrand, Johannesburg, South Africa; 51 INCAP Research Center for Prevention of Chronic Diseases, Institute of Nutrition of Central America and Panama, Guatemala City, Guatemala

**Keywords:** cardiovascular disease, prevention, aspirin, non-communicable disease

The role of aspirin in the primary prevention of atherosclerotic cardiovascular disease (CVD) is not clear, with recent clinical trials evaluating aspirin for primary prevention demonstrating both modest benefits and risks associated with its use [1]. Aspirin is not routinely recommended for primary prevention of CVD. Understanding patterns of aspirin use can inform strategies to promote guideline-concordant therapy, including de-implementation strategies to reduce unnecessary aspirin use. This study seeks to provide an updated estimate of worldwide aspirin use for primary prevention of CVD.

## Methods

**Figure 1:**
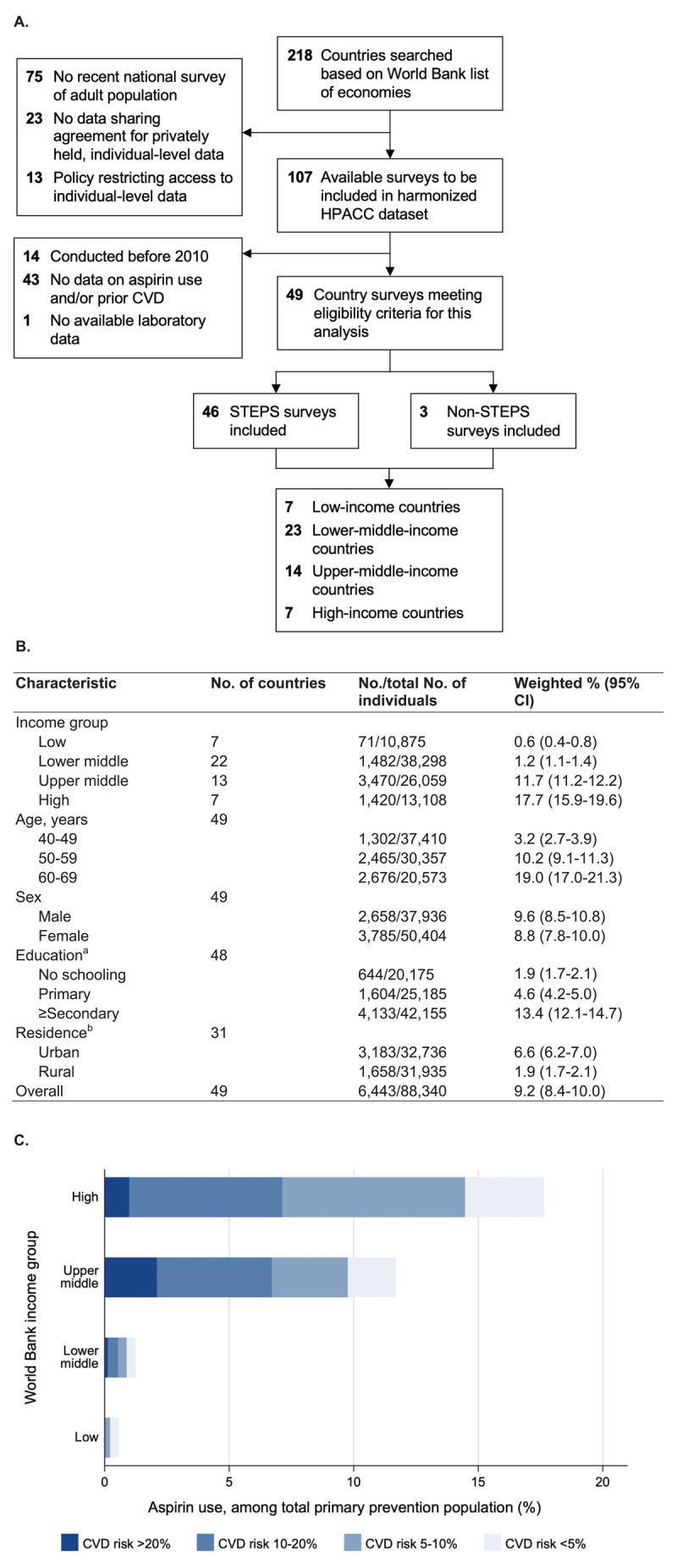
**A)** Survey inclusion flow chart. HPACC refers to The GHP Project on Access to Care for Cardiometabolic Diseases Collaboration. STEPS refers to STEPwise approach to NCD risk factor surveillance surveys. **B)** Use of aspirin for primary prevention of cardiovascular disease by income group, age, sex, education, and rurality. Estimates are weighted by each country’s 2019 population of individuals aged 40 to 69 years. Income group refers to World Bank per capita income categories in the year the survey was conducted. ^a^Education was unavailable in the survey from Tokelau. ^b^Urban vs rural residence was unavailable in the surveys from Bermuda, Botswana, Brunei, Ecuador, Eswatini, Kiribati, Kuwait, Lebanon, Myanmar, Nauru, Solomon Islands, Sri Lanka, St Vincent and the Grenadines, Tajikistan, Timor-Leste, Tokelau, Tuvalu, and the US. **C)** CVD risk categories of individuals using aspirin for primary prevention by income group. Estimates are weighted by each country’s 2019 population of individuals aged 40 to 69 years. Income group refers to World Bank per capita income categories in the year the survey was conducted. Categories of 10-year predicted CVD risk are calculated using the 2019 regionally calibrated WHO laboratory-based risk-prediction models.

We analyzed pooled individual-participant data from nationally representative health surveys conducted in 49 countries between 2013 and 2020. Details of the study design, data sources, and sample population have been described (Figure 1) [2].

The primary outcome was the use of aspirin for primary prevention of CVD. We defined primary prevention as the population without a self-reported history of myocardial infarction, stroke, or angina. We classified participants using aspirin for primary prevention into categories based on 10-year predicted CVD risk: <5%, 5–10%, 10–20%, and >20%, aligning with established guidelines [1]. The analysis used the 2019 regionally calibrated WHO laboratory-based risk-prediction models, which includes input variables of age, sex, smoking status, systolic blood pressure, history of diabetes, and total cholesterol [3].

We estimated the proportion of individuals using aspirin for the primary prevention of CVD by World Bank per-capita income categories [4]. We accounted for the complex survey design in analyses and used sampling weights rescaled in proportion to each country’s 2019 population. We conducted our analysis in Stata version 17.0 (StataCorp) and R version 4.2.2 (R Foundation). Our study was considered exempt from regulation by the institutional review board at the University of Michigan (HUM00201307).

## Results

The dataset encompassed 96,914 individuals from 49 countries, with 88,340 categorized as belonging to the primary prevention group (Figure 1). Among individuals without a self-reported history of CVD, aspirin use in the overall pooled sample was 9.2% (95% CI: 8.4%–10.0%). The prevalence of aspirin use varied significantly by income group, with estimates of 0.6% (95% CI: 0.4%–0.8%) in low-income countries, 1.2% (95% CI: 1.1%–1.4%) in lower-middle-income countries, 11.7% (95% CI: 11.2%–12.2%) in upper-middle-income countries, and 17.7% (95% CI: 15.9%–19.6%) in high-income countries (Figure 1).

A substantial proportion of individuals in higher-income countries who used aspirin for primary prevention had low predicted CVD risk. For example, among those using aspirin for primary prevention in high-income countries, 59.6% (95 CI: 55.3%–63.7%) had 10-year CVD risk less than 10% (Figure 1). The median 10-year CVD risk among individuals using aspirin for primary prevention in high-income countries was 6.2% (IQR: 3.3%–10.2%).

## Discussion

In a diverse sample of nationally representative surveys conducted between 2013–2020, we found many people using aspirin for primary CVD prevention across the world. Particularly in upper-middle-income and high-income countries, people using aspirin for primary CVD prevention have low CVD risk. This study offers the most extensive and contemporary understanding of global aspirin use among individuals without a prior history of CVD. The finding is in contrast to evidence that questions the routine use of aspirin in populations effectively managed with cholesterol and blood pressure-lowering medications [5]. A modeling study suggested that primary prevention aspirin users who are at low risk or aged greater than 60 years experience minimal or even negative lifetime net benefit as measured by quality-adjusted life-years and total life-years [6]. In this context, the current study suggests overuse of aspirin among populations without a history of CVD is an important worldwide public health problem with particular salience in upper-middle-income and high-income countries where resources may be limited and greater health system efficiency could be achieved. However, the use of aspirin for primary prevention should be contextualized within individual health systems, considering factors such as population-level CVD risk and the accessibility of cholesterol and blood pressure–lowering medications and aspirin.

Limitations of the study include the use of self-reported data on aspirin use and CVD history and the incorporation of some surveys completed before the release of recent aspirin primary prevention trials. Nevertheless, the data remain relevant, as it takes decades for medical practices to change with the evolving evidence regarding the role of aspirin in prevention [7]. Additionally, it was not possible to define with certainty whether an individual’s aspirin use was appropriate. For primary prevention, we assumed individuals to be at low CVD risk using a 10-year threshold of <10%. Finally, the 2019 WHO CVD risk equations used in our analysis are the most updated risk-prediction model for worldwide use, but the models have not been externally validated in all countries, given a paucity of CVD cohorts outside of high-income countries.

## Conclusions

Aspirin use for primary prevention varies widely globally. Strategies aimed at de-prescribing aspirin in populations unlikely to benefit should be developed, evaluated, and implemented to mitigate the potential harms associated with unnecessary aspirin use.
